# Editorial

**Published:** 1898-10

**Authors:** 


### EDITORIAL DEPARTMENT.

F. E. DANIEL, M. D., Editor.
S. E. HUDSON, M. D., Managing Editor.
A. J. SMITH. M. D., Galveston, Associate Editor.

Published Monthly at Austin, Texas, by Drs. Daniel and Hudson. Subscription price $1.00 a year in advance.

Eastern Representatives: Messrs. Monihan & Fairchild, St. Paul Building, 220 Broadway, New York City.

Official organ of the West Texas Medical Association, the Houston District Medical Association, the Austin District Medical Society, the Brazos Valley Medical Association, the Galveston County Medical Society, and several others.

### EDITORIAL.

THE YELLOW FEVER SITUATION.—At this writing, October 3d, there are nine *foci* of infection in Louisiana, to-wit: New Orleans, Franklin, Houma, Plaquemine, Clinton, Wilson, Baton Rouge, DeLoamy, and Harvey's Canal; and in Mississippi there are seven, to-wit: Jackson, Taylor, Orwood, Oxford, Harrison, Poplarville and Waterford. Of course Texas is quarantined against all these places. There were reported up to October 1st, 276 cases in Louisiana, and fourteen deaths; about five per cent. At Oxford, Mississippi, the percentage has been over thirty; at other points less than two per cent. The fever is said to be of a "mild type," whatever that may mean. I don't believe there can be a "mild type" of yellow fever, any more than there can be a mild kind of strychnine, for instance. Yellow fever is produced by a bacillus, a poison, and it is a matter of how much poison one gets in, in reference to the power of his phagocytes to tackle and dispose of it. If the bacillus is not as strong as in years past, why? What attenuated it? The very low mortality in many places this and last year—so low in fact as to throw a doubt upon the correctness of the diagnosis, is surely not attributable to a better knowledge of the disease and its proper treatment by those to whom the treatment falls, the local profession, and it would seem that little progress has been made in sanitary methods looking to limiting the contagion. It *got out* at Franklin, from one case, promptly diagnosed and taken in hand by the Marine Hospital Service and Louisiana Board of Health, notwithstanding the house and all infected things were burned, the burning extending "a foot deep in the ground," so said; and from one case August 12th, there resulted one hundred and seventy-five cases by October 1st. It spread also from Harvey's Canal to Clinton, Baton Rouge, Wilson, and Plaquemine. How it got to Harvey's, deponent sayeth not. There is none in Texas. As all of October is likely to be hot, there is no telling at this time to what points the fever may spread, or to what extent it may prevail.

---

A SUBSCRIBER at Fort Worth, who in writing us requests that his name be not used, says:

"In upholding you in your efforts to elevate the practice of medicine in Texas, I meet with much opposition to the proposed new law on the subject of regulating the practice, or any new law, in fact. The doctors of the country and small villages who are graduates, and who have registered as required by law, fear that any new law would work a hardship on them. They say they do not actively oppose the proposed law, but they fear that should it pass it will entail additional expense on them and subject them to another examination. They fear they might fail to pass examination in the rudiments of some branches, etc. I have stated in reply to such objections that those who have qualified under the existing law, or any previous law, are exempt from examination. Am I right?"

[Eminently so; no law can be made retroactive. Most laws "take effect from and after passage." No law can take effect before its passage.—ED.]

